# Isolation of Glycinin (11S) from Lipid-Reduced Soybean Flour: Effect of Processing Conditions on Yields and Purity

**DOI:** 10.3390/molecules17032968

**Published:** 2012-03-09

**Authors:** Kequan Deng, Youru Huang, Yufei Hua

**Affiliations:** 1 State Key Laboratory of Food Science and Technology, School of Food Science and Technology, Jiangnan University, 1800 Lihu Avenue, Wuxi 214122, Jiangsu, China; Email: dengkq@sina.cn; 2 School of Biological Science and Food Engineering, Changshu Institute of Technology, Changshu 215500, Jiangsu, China; Email: huangyouru@yahoo.com.cn

**Keywords:** glycinin, isolation, pH, reducing agent, store time

## Abstract

Defatted soybean flour was treated with hexane and ethanol to reduce lipid content and heated to inactivate lipoxygenase (LOX, linoleate:oxygen reductase; EC 1.13.11.12) to obtain lipid-reduced soybean flour (LRSF). The effects of processing conditions such as pH, reducing agent and storage time on yields and purity of glycinin (11S) were evaluated in the fractionation of soybean glycinin isolated from LRSF. Adjusting the pH of protein extract from 6.2 to 6.6, the yield of glycinin decreased by 16.71%, while the purity of the protein increased by 4.60%. Sulfhydryl and disulfide content of proteins increased by degrees with increasing pH. Compared with dithiothreitol (DTT) or β-mercaptoethanol (ME) as reducing agent, the yield of glycinin was the highest when sodium bisulfite (SBS) was added to the protein extract at pH 6.4. The effect of DTT on yields of glycinin was the lowest of the three kinds of reducing agent. The purity of glycinin was similar when the three kinds of reducing agent were used. These results showed that SBS was the best choice for the isolation of 11S-rich fraction. Prolonging storage time in the precipitation stage, 10 h was the best for yields and purity of glycinin in the experiment, while there was no significant difference at *P* ≥ 0.05 for total sulfhydryl and disulfide content. The decreased free sulfhydryl content of glycinin indicated that the oxidation of free sulfhydryls and the formation of disulfide bonds occurred when the extraction time was prolonged.

## 1. Introduction

Soybean proteins are composed of two major components, glycinin and β-conglycinin, which account for around 40% and 30% of total proteins, respectively [1]. Glycinin, a globulin storage protein of soybean and designated as the 11S size fraction by ultracentrifugation, consists of an acidic and a basic polypeptide linked by a single disulfide bond [2]. Each polypeptide of glycinin displays different properties that may be exploited in food applications; e.g., the acidic subunits from the 11S proteins shows superior gel-forming ability, and gel strength is increased with the content of the acidic subunits [3]. The basic subunits from the 11S proteins may be quite soluble in the pH range of acidic beverages and could represent a significant source of acid soluble proteins useful in beverages, mayonnaise and salad dressings [4]. The separation of the glycinin (11S) from soybean proteins may generate potential ingredients with improved functional properties that can be used in high-value industrial applications. Furthermore, the investigation of the acidic and basic subunits will help us to know more about the role of the 11S subunits in influencing certain industrial soy protein characteristics.

Many methods have been reported in the literature for purifying the glycinin fraction. Nearly all the current methods involve separating an initial crude glycinin fraction from an alkaline soy protein extract by precipitation in the cold at pH 6.3–7.0 [5,6,7,8,9]. However, few data have been reported on the fractionation of soybean glycinin isolated from LRSF. In previous papers [10,11], we reported that when defatted soybean flour with LOX activity were submitted to water extraction under mildly alkaline conditions in the preparation of soybean protein isolate, LOX may catalyze the oxidation of residual lipids present in soybean flour to initiate free-radical chain aggregation, causing undesirable changes in the nutritional and functional properties of the proteins. As a part of the above research, we present in this paper the effect of processing conditions on yields and purity of glycinin (11S) isolated from LRSF. Some relative variables such as pH, reducing agent and storage time are used for reference according to the literature [5,6,7,8,9]. The protein constitution was analyzed by sodium dodecyl sulfate-polyacrylamide gel electrophoresis (SDS-PAGE). The sulfhydryl and disulfide contents of isolated protein fractions were also evaluated.

## 2. Results and Discussion

### 2.1. Chemical Composition and Solubility of Starting Flour

The total protein, moisture and lipids contents in the starting flour were 56.37% (dry basis), 10.32% and 5.21%, respectively. The Protein dispersion index (PDI) value of the soy flour was 68.21%, which was determined by the AACC method [12]. Glycinin and β-conglycinin represented 53.12% and 31.37% of the total protein, respectively.

### 2.2. The Effect of pH on Yields and Purity of Glycinin (11S)

**Figure 1:**
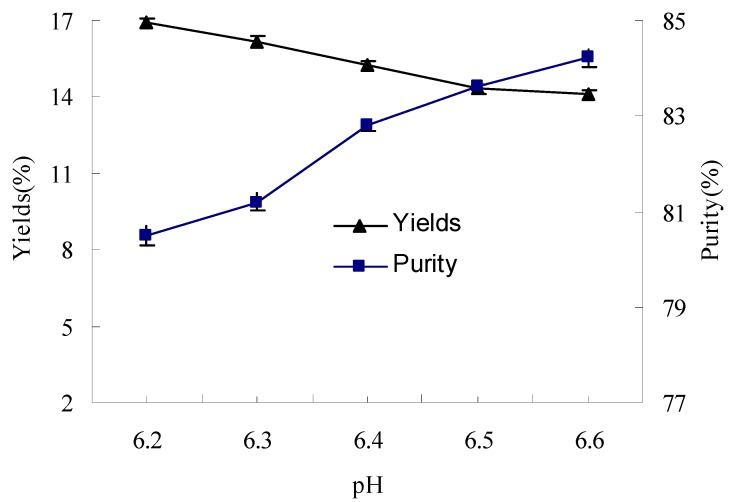
The effect of pH on yields and purity of glycinin (11S).

The effect of pH on yields and purity of glycinin (11S) was shown in Figure 1. In the protein extract, when the pH was adjusted from 6.2 to 6.6, the yield of the 11S-rich fraction decreased remarkably by 16.71%. This was attributed to the sharp decrease in protein solubility when pH increased [13]. In contrast, the purity of the 11S-rich fraction increased by 4.60% when the pH was adjusted from 6.2 to 6.6. This behavior appears to be consistent with the different pH-solubility profiles of the two globulins, namely 7S and 11S [13,14].

### 2.3. The Effect of Different Kinds of Reducing Agent on Yields and Purity of Glycinin (11S)

**Figure 2:**
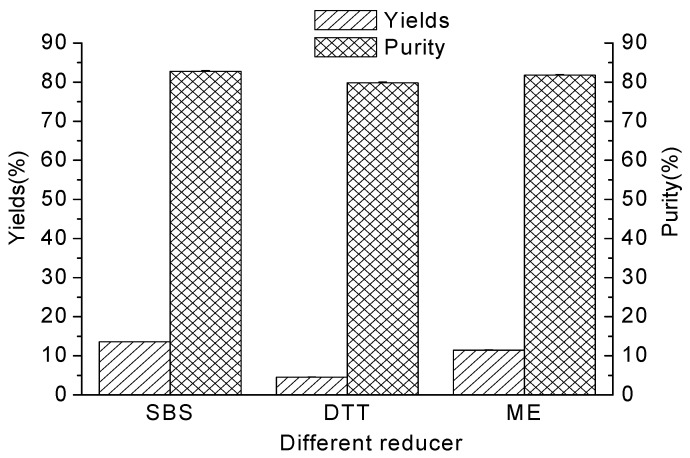
The effect of different kinds of reducing agent on yields and purity of glycinin (11S).

The reagents SBS, DTT and ME, acting on the disulfide bond of protein and causing the depolymerization of the polymeric compounds, could increase the protein solubility [15]. ME was used as the reducing agent in the method of Thanh and Shibasaki [9] and SBS was used by some researchers [16,17,18]. It was confirmed that SBS could be used instead of ME, and the protein contents of the 11S globulin fraction were 19% and 41%, respectively, for ME and SBS [7]. The effect of different kinds of reducing agent on yields and purity of glycinin (11S) is shown in Figure 2. Compared with DTT or ME reducing agent, the yield of the 11S-rich fraction was the highest when SBS was added to the protein extract at pH 6.4. Yields of glycinin (11S) when DTT was used as the reducing agent were the lowest. When DTT was added to the protein extract, the yield of the 11S-rich fraction was only 33.43% of SBS and 39.69% of ME. The purity of the 11S-rich fraction was similar when the three kinds of reducing agent were added to the protein extract, but the variance between SBS, DTT or ME was smaller than the yield. The purity of the 11S-rich fractions was 96.40% of SBS and 97.59% of ME when DTT was added to the protein extract. That is why most researchers used SBS as reducing agent in the protein extract during the preparation of 11S-rich fraction. [6,8,15,16,17,18,19,20].

### 2.4. The Effect of Slurry Storage Time on Yields and Purity of Glycinin (11S)

**Figure 3:**
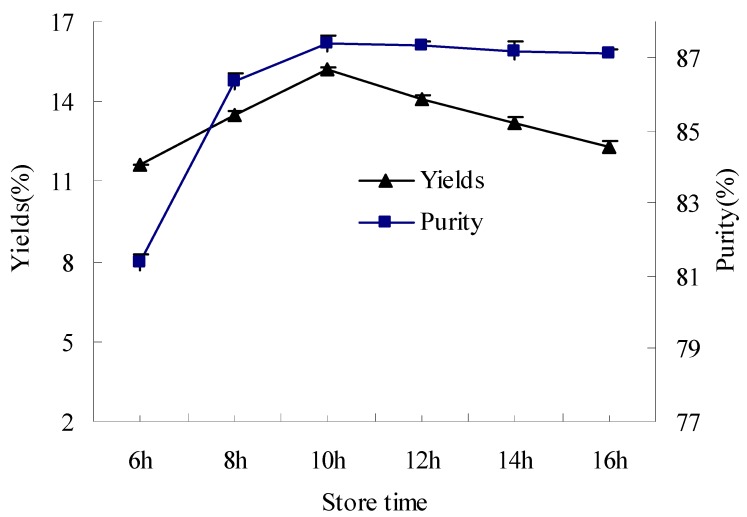
The effect of slurry storage time on yields and purity of glycinin (11S).

Wolf *et al.* reported that cryoprecipitation of soybean 11S protein was complete or nearly complete in 1–2 h [21]. But other researchers such as Deak, Wu and Hou reported that precipitation of glycinin (11S) took 12–16 h or overnight stored at 4–7 °C [6,17,18,19,20,22]. Therefore, slurry storage times ranging from 6 h to 16 h were selected in our experiment (Figure 3). With the prolonging of time, the yields of isolated 11S fractions seemed to increase at first, and then to decrease. The highest yields were obtained at 10 h. The purities of isolated 11S fraction changed little between 10 h and 16 h, but increased markedly above 6 h. It seemed that purity of glycinin (11S) could not come to a certain level until the sedimentation reached up to a specific time at 4 °C. Of all selected times, 10 h was the best for yields and purity of glycinin (11S) in the present experiments. The main reason for the decrease of yield with the prolonging time might be the depolymerization of glycinin (11S) caused by reducing agent added in the protein extraction [23].

### 2.5. SDS-PAGE of Selected Fractions

**Figure 4:**
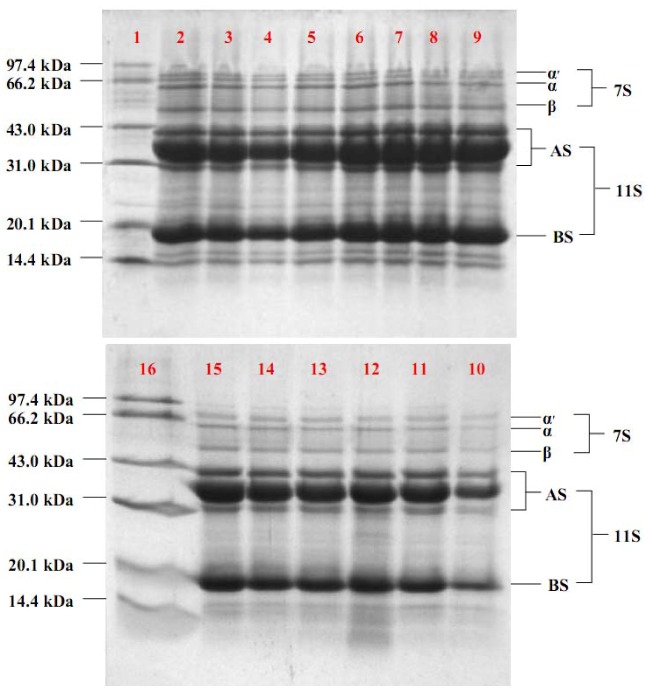
SDS-PAGE band patterns of the 11S-rich fractions obtained by different methods: 1 the molecular weight markers to which be pointed with arrows in the left; 2 pH6.2; 3 pH6.3; 4 pH6.4; 5 pH6.5; 6 pH6.6; 7 SBS; 8 DTT; 9 ME; 10 6h; 11 8h; 12 10h; 13 12h; 14 14h; 15 16h; 16 markers. AS and BS indicated acidic and basic peptides of glycinin (11S) respectively, while α', α and β indicated subunits of β-conglycinin (7S). 20 μg of each sample (protein basis) was loaded.

SDS-PAGE was used to examine the purity of different fractions obtained by different methods (Figure 4). The major contaminant of the 11S-rich fraction obtained at pH 6.2–6.6 (lanes 2 to 6) or that precipitated at pH 6.4 with SBS, DTT, or ME (lanes 7, 8 and 9) was subunits of β-conglycinin, namely α', α and β. At pH 6.4, the addition of SBS, DTT, or ME induced a β-conglycinin contamination in the 11S-rich fraction as shown in lanes 7–9. Reducing agent decreased the purity of the 11S-rich fraction at pH 6.4 (lanes 7, 8 and 9, compared with lanes 11–15). The relative quantities of the 11S globulin in lanes 11–15 were higher than those in lanes 2–9. These results suggested that the purity of the 11S-rich fraction would be improved by reducing the relative contents of β-conglycinin with prolonged time.

### 2.6. Sulfhydryl and Disulfide Content of Proteins

Sulfhydryl and disulfide bonds of proteins play an important role in protein structure and functionality. Even though soybean varietal differences may account for the variability of sulfhydryl values, the protein preparation method is the major cause of sulfhydryl variability [19,24], especially when adding different kinds of reducing agent such as SBS, DTT or ME during extraction. Sulfhydryl and disulfide content of proteins are shown in Table 1.

**Table 1:** Sulfhydryl and disulfide content of proteins ^a^.

Sample		Free SH ^b^	Total SS and SH ^b^
pH	6.2	5.85 (0.08) ^c^A ^d^	50.12 (0.02)A
	6.3	6.01 (0.04)A	50.57 (0.08)B
	6.4	6.35 (0.07)B	50.96 (0.09)C
	6.5	6.63 (0.03)C	51.20 (0.08)C
	6.6	6.87 (0.03)E	51.73 (0.09)D
reducer	SBS	6.39 (0.04)B	50.82 (0.43)C
	DTT	4.38 (0.02)F	47.31 (0.36)E
	ME	5.73 (0.04)A	50.03 (0.35)A
store time	6 h	5.77 (0.06)A	50.19 (0.33)A
	8 h	6.23 (0.04)B	51.26 (0.13)C
	10 h	6.72 (0.02)C	51.53 (0.19)C
	12 h	6.76 (0.05)C	51.46 (0.25)C
	14 h	6.50 (0.05)BD	51.37 (0.19)C
	16 h	6.30 (0.05)B	51.18 (0.24)C

^a^ All values are the mean of triplicate determinations; ^b^ μmoles SH/g protein; ^c^ Values in parenthesis are standard errors; ^d^ The same letter in columns indicates no significant difference at *P* ≥ 0.05.

When the extract pH was increased from 6.2 to 6.6, sulfhydryl and disulfide content of proteins increased by degrees and the variance of sulfhydryl and disulfide content between samples showed significant differences at the 0.05 level. When SBS was added to the protein extract, the content of sulfhydryl and disulfide groups was markedly different compared to the other two kinds of reducing agent. Compared with pH 6.4 extracted sample, SBS showed no significant difference at the 0.05 level. When the storage time of the protein extract was prolonged from 8–16 h, there was no significant difference at *P* ≥ 0.05 for total sulfhydryl and disulfide content. However, free sulfhydryl content of three samples (pH 6.4, reducer SBS, and storage time 12 h) showed significant differences at the 0.05 level. The reason for this is not clear. The tendency of decrease with prolonged extraction time (12–16 h) indicated that the oxidation of free sulfhydryl and the formation of disulfide bond ocurred.

## 3. Experimental

### 3.1. Materials

Low-denatured, defatted soybean flour was provided by Shandong Wandefu Industrial & Commercial Co., Ltd. All other reagents and chemicals were of analytical grade.

### 3.2. The Treatment of Defatted Soybean Flour

Defatted soybean flour, which had been ground to pass 80 mesh screens, was extracted with hexane and ethanol with ratio of 1:2:4 (W/W) at 20 °C for 1.0 h. The slurry was vacuum filtered and the filter cake was washed with 95% ethanol at 20 °C for 1.0 h with a flour to solvent ratio of 1:5 (W/W). After vacuum filtration, the cake was vacuum dried under room temperature. The dried material was ground to pass 80 mesh, vacuum heated at 90 °C for 30 min to reinforce LOX inactivation and lipid-reduced soybean flour (LRSF) thus obtained.

### 3.3. The Separation of 11S Fraction

**Figure 5:**
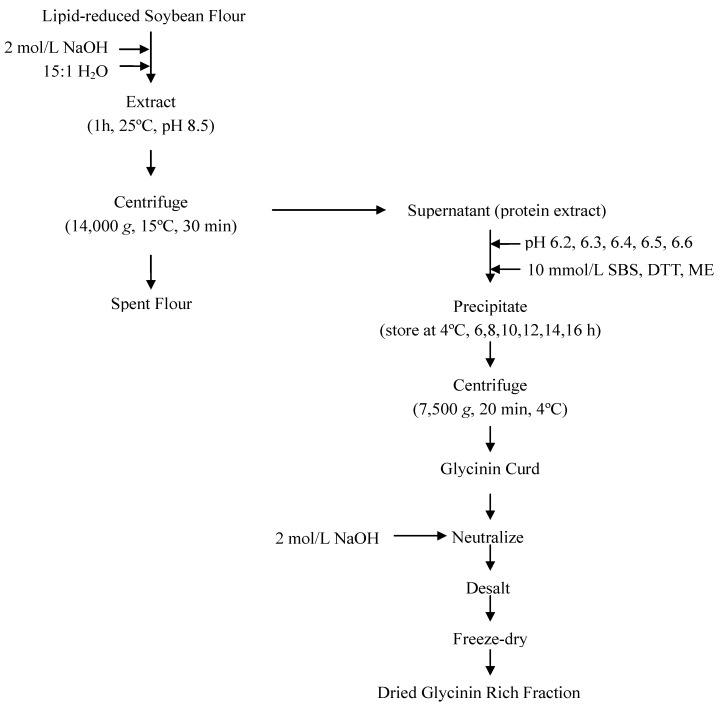
Flow diagram for the procedure of fractionating soybean glycinin (11S).

The control fractionating procedure was Deak’s method [6] with minor modification (Figure 5). About 100 g lipid-reduced soybean flour (LRSF) was extracted with deionized water at 15:1 (W/W) water-to-flour ratio and the pH was adjusted to 8.5 with 2 mol/L NaOH. The slurry was stirred for 1 h and centrifuged at 14,000 *g* and 15 °C for 30 min. The protein extract was decanted and sufficient NaHSO_3_ was added to the protein extract to achieve 10 mmol/L SO_2_. The pH was adjusted to 6.4 with 2 mol/L HCl. The slurry was stored at 4 °C for 12 h and then centrifuged at 7,500 *g* and 4 °C for 20 min. A glycinin-rich fraction was obtained as the precipitated curd. This fraction was redissolved in deionized water, adjusted to pH 7 with 2 mol/L NaOH, desalted, and freeze-dried.

### 3.4. The Effect of pH in the Presence of Sodium Bisulfite (SBS)

This experiment studied the influence of 10 mmol/L SO_2_ at various pH values (from 6.2 to 6.6) in the first precipitation step (Figure 5). Other steps were the same as the control fractionating procedure. Each experiment was at least duplicated.

### 3.5. The Effect of Different Kinds of Reducing Agent in the pH 6.4

This experiment evaluated the effect of different kinds of reducing agent such as sodium bisulfite (SBS), dithiothreitol (DTT) or β-mercaptoethanol (ME) (at 10 mmol/L) on the fractionation (Figure 5). The pH of the slurry was adjusted to 6.4 with 2 mol/L HCl and stored at 4 °C for 12 h and then centrifuged at 7,500 g and 4 °C for 20 min. Other steps were the same as the control fractionating procedure. Each experiment was at least duplicated.

### 3.6. The Effect of Slurry Store Time in the Presence of Sodium Bisulfite (SBS)

This experiment assessed the efficiency of separation as influenced by slurry storage time in the presence of sodium bisulfite (SBS) (Figure 5). The protein extract was decanted and sufficient NaHSO_3_ was added to the protein extract to achieve 10 mmol/L SO_2_. The pH was adjusted to 6.4 with 2 mol/L HCl. The slurry was stored at 4 °C for 6–16 h and then centrifuged at 7,500 g and 4 °C for 20 min. Other steps were the same as the control fractionating procedure. Each experiment was at least duplicated.

### 3.7. Analysis of Protein Constitution by SDS-PAGE

SDS-PAGE was carried out at a constant current (20 mA) in a vertical slab unit (Beijing Liuyi Instrument Factory, Beijing, China) according to the method of Weber and Osborn [25]. Stacking gel was 4% acrylamide, 0.32% *N*,*N*’-methylene-bis-acrylamide in 0.125 mol/L Tris-HCl buffer, pH 6.8, and running gel was 12% acrylamide, 0.27% *N*,*N*’-methylene-bis-acrylamide in 0.43 mol/L Tris-HCl buffer, pH 8.9. Both gels contained 0.1% SDS. The electrode buffer was 0.05 mol/L Tris-0.384 mol/L glycine buffer, pH 8.3, containing 0.1% SDS. Soybean protein samples were mixed with reductive sample buffer (2% SDS, 5% β-mercaptoethanol, 10% glycerol, 0.02% bromophenol blue, 0.01 mol/L Tris-HCl buffer, pH 8.0) to give a concentration of 2 mg/mL and the solution was centrifuged at 1,500 g for 10 min to get the supernatant as sample solution. To each cell, 10 μL sample solution was loaded. Gel slabs were fixed in methanol-acetic acid-water (45:10:45, V/V) for 12.0 h and stained using the Coomassie brilliant blue G-250 stain solution (40% methanol, 10% acetic acid, 0.1% Coomassie brilliant blue G-250) for 1.0 h and destained by destain solution (acetic acid-water, 3:37, V/V) for 6.0 h with 2–3 changes of destain solution. The band patterns were then photographed and analyzed with Quantity One software version 4.4 (Bio-Rad Laboratories Inc., Hercules, CA, USA). The purity of a single fraction was calculated according to Deak *et al.* [6]. Fraction purity (%) = (summed light intensity of bands corresponding to a designated fraction) / (summed light intensity of all bands) × 100. Glycinin and β-conglycinin subunit bands were confirmed by using purified standards produced according to the methods of O’Keefe *et al.* [26]. All measurements were at least duplicated.

### 3.8. Sulfhydryl and Disulfide Content of Proteins

The sulfhydryl (free and buried SH) and total sulfhydryl (SH and reduced SS) groups of proteins were determined by titration with 5,5'-Dithiobis(2-nitrobenzoate) (DTNB) (Sigma Chemical Co., St. Louis, MO, USA), using the general procedure of Ellman [27]. Soybean protein samples (75 mg) were suspended in 10 mL 0.1 mol/L phosphate buffer (pH 8.0), l mmol/L EDTA and 1% sodium dodecyl sulfate by stirring with a 1.25 cm stir bar in a 20 mL beaker on medium speed for 30 min at 20 °C.

For SH determinations, to 3 mL of protein solution, 3 mL of 0.l mol/L phosphate buffer with EDTA and SDS and 0.1 mL DTNB reagent were added, vortexed, and incubated at 25 °C for 1.0 h [28]. This mixture was centrifuged at 10,000 g for 30 min. 

For total SH determination, the method of Beveridge, Toma and Nakai was modified by using 0.1 mol/L phosphate buffer (pH 8.0), l mmol/L EDTA, and 1% SDS instead of Tris-glycine buffer and 0.08 mL of DTNB reagent was added [29]. That is, 1 mL of protein solution was added to 0.05 mL of 2-mercaptoethanol and 4 mL of Urea-GuHCl and the mixture was incubated for 1.0 h at 25 °C. After an additional 1.0 h incubation with 10 mL of 12% TCA, the tubes were centrifuged at 5,000 g in an Anke centrifuge (TGL-16B Model, Shanghai Anting Scientific Instrument Factory, Shanghai, China) for 10 min. The precipitate was twice resuspended in 5 mL of 12% TCA and centrifuged to remove 2-mercaptoethanol. The precipitate was dissolved in 10 mL of 0.1 mol/L phosphate buffer (pH 8.0), l mmol/L EDTA, and 1% SDS and the color was developed with 0.08 mL of Ellman’s reagent. 

Absorbance was measured at 412 nm *vs*. reagent blanks. Calculations were based on extinction coefficient of 13,600 M^−^^1^ cm^−^^1^ for the thiolate chromogen [29] according to the following equation:

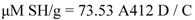

where A412 ＝ the absorbance at 412 nm; C = the sample concentration in mg solids / mL; D ＝ the dilution factor, 2.03 and 10.08 for SH and total SH (SH + reduced SS), respectively; and 73.53 is derived from 106 / (1.36 × 104); 1.36 × 104 is the molar absorptivity [27] and 106 is for conversions from the molar basis to the μM / mL basis and from mg solids to g solids.

### 3.9. Statistics

All the data were averaged using Microsoft Excel 2007. Analysis of variance (ANOVA) was performed using Origin 7.5 (OriginLab Corp., Northampton, MA, USA).

## 4. Conclusions

The glycinin fractions were mostly purified from defatted soybean flour [5,6,7,8,9]. However, we found that lipid remained in the soybean flour and processing conditions of water extraction had an effect on the quality of the isolated protein, so we undertook several measures to investigate the lipid-protein interactions in the protein extraction, such as decreasing lipid content of defatted soybean flour, inactivating LOX activity and changing protein extraction conditions. In the present study, the effects of processing conditions such as pH, reducing agent and storage time on yields and purity of glycinin (11S) were evaluated in the fractionation of soybean glycinin isolated from LRSF. pH values played an important role in the isolation process by influencing the protein solubility [13,14,23]. By adjusting the pH of protein extract from 6.2 to 6.6, the yield of the 11S-rich fraction decreased significantly by 16.71%, while the purity of the 11S-rich fraction increased only by 4.60%. It is interesting to note that, when the extract pH was increased from 6.2 to 6.6, the sulfhydryl and disulfide group content of proteins increased by degrees, and the variance of sulfhydryl and disulfide content between samples showed significant differences at the 0.05 level. The reason for this increase in the sulfhydryl and disulfide content of proteins with pH was not clear and is worth investigating further. That was why many researchers chose pH 6.4 for the purity of glycinin (11S) during protein extraction [6,7,9,17,20]. 

The substitution of different kinds of reducing agent for fractionation of soybean 11S globulins was also performed in the present study. When SBS was added to the protein extract, the content of sulfhydryl and disulfide groups was markedly different from those seen with the other two kinds of reducing agent. Compared with DTT or ME reducing agent, the yield of the 11S-rich fraction was the highest when SBS was added to the protein extract at pH 6.4. DTT produced the lowest yields of glycinin (11S) among the three kinds of reducing agent. When DTT was added to the protein extract, the yield of the 11S-rich fraction was only 33.43% of SBS and 39.69% of ME respectively. The purity of the 11S-rich fraction was similar when the three kinds of reducing agent were added to the protein extract, but the variance between SBS, DTT or ME was smaller than the yield change. The purity of the 11S-rich fraction was 96.40% of SBS and 97.59% of ME when DTT was added to the protein extract. These results demonstrated that if we had to optimize both yield and purity at the same time, SBS would be the best choice of reducing agent for the isolation of 11S-rich fraction.

When prolonging storage time in the precipitation stage, the yields of isolated 11S fractions seemed to increase at first, and then to decrease. The highest yields were obtained at 10 h. The purities of isolated 11S fraction changed little between 10 h and 16 h, but markedly increased above 6 h. Of all selected times, 10 h was the best for yields and purity of glycinin (11S) in the experiments. For sulfhydryl and disulfide content, there was no significant difference at *P* ≥ 0.05 when the storage time of the protein extract was prolonged from 8–16 h. The decreasing tendency of free sulfhydryl groups with prolonged extraction time (12–16 h) indicated the oxidation of free sulfhydryls and the formation of disulfide bonds.

## References

[1] Utsumi S., Matsumura Y., Mori T., Damodaran S., Paraf A. (1997). Structure-Function Relationships of Soy Proteins. Food Proteins and Their Application.

[2] Delwiche S.R., Pordesimo L.O., Panthee D.R., Pantalone V.R. (2007). Assessing glycinin (11S) and β-conglycinin (7S) fractions of soybean storage protein by near-infrared spectroscopy. J. Am. Oil Chem. Soc..

[3] Fukushima D. (2001). Recent progress in research and technology on soybeans. Food Sci. Technol. Res..

[4] Kinsella J.E. (1979). Functional properties of soy proteins. J. Am. Oil Chem. Soc..

[5] Brooks J.R., Morr C.V. (1985). Current aspects of soy protein fractionation and nomenclature. J. Am. Oil Chem. Soc..

[6] Deak N.A., Murphy P.A., Johnson L.A. (2006). Effects of NaCl concentration on salting-in and dilution during salting-out on soy protein fractionation. J. Food Sci..

[7] Nagano T., Hirotsuka M., Mori H., Kohyama K., Nishinari K. (1992). Dynamic viscoelastic study on the gelation of 7S globulin from soybeans. J. Agric. Food Chem..

[8] Teng Z., Liu C., Yang X.Q., Li L., Tang C.H., Jiang Y.M. (2009). Fractionation of soybean globulins using Ca^2+^ and Mg^2+^: A comparative analysis. J. Am. Oil Chem. Soc..

[9] Thanh V.H., Shibasaki K. (1976). Major proteins of soybean seeds. A straightforward fractionation and their characterization. J. Agric. Food Chem..

[10] Huang Y.R., Hua Y.F., Qiu A.Y. (2006). Soybean protein aggregation induced by lipoxygenase catalyzed linoleic acid oxidation. Food Res. Int..

[11] Huang Y.R., Hua Y.F., Qiu A.Y. (2006). Detection of free radical transfer in lipoxygenase I-B-catalyzed linoleic acid-soybean proteins interaction by electron spin resonance spectroscopy (ESR). J. Agric. Food Chem..

[12] AACC. *“AACC Approved Methods,”* rev. American Association of Cereal Chemists: St. Paul, MN, USA, 1975.

[13] Yuan Y.J., Velev O.D., Chen K., Campbell B.E., Kaler E.W., Lenhoff A.M. (2002). Effect of pH and Ca^2+^ induced associations of soybean proteins. J. Agric. Food Chem..

[14] Lakemond C.M.M., De Jongh H.H.J., Hessing M., Gruppen H., Voragen A.G.J. (2000). Soy glycinin: Influence of pH and ionic strength on solubility and molecular structure at ambient temperatures. J. Agric. Food Chem..

[15] Liu C., Wang H.L., Cui Z.M., He X.L., Wang X.S., Zeng X.X., Ma H. (2007). Optimization of extraction and isolation for 11S and 7S globulins of soybean seed storage protein. Food Chem..

[16] Silvana P., Maria C.A. (1995). Soy protein isolate components and their interactions. J. Agric. Food Chem..

[17] Wu S.W., Murphy P.A., Johnson L.A., Fratzke A.R., Reuber M.A. (1999). Pilot-plant fractionation of soybean glycinin and β-conglycinin. J. Am. Oil Chem. Soc..

[18] Wu S.W., Murphy P.A., Johnson L.A., Reuber M.A., Fratzke A.R. (2000). Simplified process for soybean glycinin and β-conglycinin fractionation. J. Agric. Food Chem..

[19] Deak N.A., Murphy P.A., Johnson L.A. (2006). Effects of reducing agent concentration on soy protein fractionation and functionality. J. Food Sci..

[20] Deak N.A., Murphy P.A., Johnson L.A. (2007). Characterization of fractionated soy proteins produced by a new simplified procedure. J. Am. Oil Chem. Soc..

[21] Wolf W.J., Sly D.A. (1967). Cryoprecipitation of soybean 11S protein. Cereal Chem..

[22] Hou D.H.J., Chang S.K.C. (2004). Structural characteristics of purified glycinin from soybeans stored under various conditions. J. Agric. Food Chem..

[23] Lui D.Y.M., White E.T., Litster J.D. (2007). Dissolution behavior of soy proteins and effect of initial concentration. J. Agric. Food Chem..

[24] Wolf W.J. (1993). Sulfhydryl content of glycinin: Effect of reducing agents. J. Agric. Food Chem..

[25] Weber K., Osborn M.J. (1969). The reliability of molecular weight determinations by sodium dodecyl sulfate polyacrylamide gel electrophoresis. J. Agric. Food Chem..

[26] O’Keefe S.F., Wilson L.A., Resurreccion A.P., Murphy P.A. (1991). Determination of the binding of hexanal to soy glycinin and betaconglycinin in an aqueous model system using a headspace technique. J. Agric. Food Chem..

[27] Ellman G.L. (1959). Tissue sulfhydryl groups. Arch. Biochem. Biophys..

[28] Boatright W.L., Hettiarachchy N.S. (1995). Effect of lipids on soy protein isolate solubility. J. Am. Oil Chem. Soc..

[29] Beveridge T., Toma S.J., Nakai S. (1974). Determination of SH- and SS-groups in some food proteins using Ellman’s reagent. J. Food Sci..

